# Circulating microRNAs in Sera Correlate with Soluble Biomarkers of Immune Activation but Do Not Predict Mortality in ART Treated Individuals with HIV-1 Infection: A Case Control Study

**DOI:** 10.1371/journal.pone.0139981

**Published:** 2015-10-14

**Authors:** Daniel D. Murray, Kazuo Suzuki, Matthew Law, Jonel Trebicka, Jacquie Neuhaus, Deborah Wentworth, Margaret Johnson, Michael J. Vjecha, Anthony D. Kelleher, Sean Emery, INSIGHT ESPRIT and SMART Study Groups

**Affiliations:** 1 The Kirby Institute for Infection and Immunity in Society, University of New South Wales, Sydney, Australia; 2 Department of Internal Medicine, University of Bonn, Bonn, Germany; 3 University of Minnesota, Minneapolis, Minnesota, United States of America; 4 Ian Charleson Day Centre, Royal Free Hampstead NHS Trust, London, United Kingdom; 5 Institute for Clinical Research, Veterans Affairs Medical Center, Washington D.C., United States of America; FIOCRUZ, BRAZIL

## Abstract

**Introduction:**

The use of anti-retroviral therapy (ART) has dramatically reduced HIV-1 associated morbidity and mortality. However, HIV-1 infected individuals have increased rates of morbidity and mortality compared to the non-HIV-1 infected population and this appears to be related to end-organ diseases collectively referred to as Serious Non-AIDS Events (SNAEs). Circulating miRNAs are reported as promising biomarkers for a number of human disease conditions including those that constitute SNAEs. Our study sought to investigate the potential of selected miRNAs in predicting mortality in HIV-1 infected ART treated individuals.

**Materials and Methods:**

A set of miRNAs was chosen based on published associations with human disease conditions that constitute SNAEs. This case: control study compared 126 cases (individuals who died whilst on therapy), and 247 matched controls (individuals who remained alive). Cases and controls were ART treated participants of two pivotal HIV-1 trials. The relative abundance of each miRNA in serum was measured, by RTqPCR. Associations with mortality (all-cause, cardiovascular and malignancy) were assessed by logistic regression analysis. Correlations between miRNAs and CD4+ T cell count, hs-CRP, IL-6 and D-dimer were also assessed.

**Results:**

None of the selected miRNAs was associated with all-cause, cardiovascular or malignancy mortality. The levels of three miRNAs (miRs -21, -122 and -200a) correlated with IL-6 while miR-21 also correlated with D-dimer. Additionally, the abundance of miRs -31, -150 and -223, correlated with baseline CD4+ T cell count while the same three miRNAs plus miR-145 correlated with nadir CD4+ T cell count.

**Discussion:**

No associations with mortality were found with any circulating miRNA studied. These results cast doubt onto the effectiveness of circulating miRNA as early predictors of mortality or the major underlying diseases that contribute to mortality in participants treated for HIV-1 infection.

## Introduction

While HIV-1 infection remains a critical problem, the introduction of effective anti-retroviral drugs has been associated with dramatic decreases in both the prevalence of AIDS and the resulting morbidity and mortality, particularly in the developed world. Current estimates suggest that up to 12.9 million people are receiving some form of anti-retroviral therapy (ART)[1]. However, there is currently no cure and treatment remains life-long. People on long term ART have increased rates of morbidity and mortality compared to uninfected individuals and these differences appear related to a collection of end organ diseases that collectively have been termed Serious Non-AIDS Events (SNAEs) [2–5].

The cause of these SNAEs seems to be multi-factorial. Aging [6], high-risk behaviour [7], co-infections [8], ART toxicity [9], CD4+ T cell depletion [10, 11], microbial translocation [12] and immune activation [13–19] all appear to contribute to SNAE pathogenesis. In the ART-treated HIV-1 infected control arm participants in the large, well-characterized trials SMART and ESPRIT [20–23] baseline levels of the soluble markers hs-CRP, D-dimer and IL-6 as well as CD4+ T cell count showed strong associations with mortality [24–26]. However, the exact mechanisms that lead to SNAEs are still unclear. How hs-CRP, D-dimer, IL-6 and CD4+ T cell number affect their progression is equally as uncertain. While these biomarkers associate with SNAEs the strength of these associations are lower than required for clinical utility [26]. Further research is essential to find new biomarkers in order to strengthen the predictive power of the established biomarkers and to provide further insight into the exact role they play in the aetiology of SNAEs and mortality.

MicroRNAs (miRNAs) are small non-coding strands of RNA that act as important post-transcriptional regulators of gene expression. They act upon complimentary strands of mRNA inhibiting protein production in the cytoplasm. In addition to their cellular function, miRNAs have also recently been found to be abundant in the circulation. These circulating miRNAs are suspected to play an important role in intercellular communication and hold promise as non-invasive biomarkers in a number of disease conditions [27]. The area of circulating miRNAs is a relatively new field as it was originally thought that the high levels of RNases in the circulation would prevent their existence [28]. However, miRNAs are extremely stable and able to avoid RNase activity through two methods: first, by associating with argonaute (AGO) proteins (>90% of circulating miRNAs appear to be AGO bound) [29, 30]; and second, by being secreted in exosomes [30, 31]. It is thought that the miRNAs bound to AGO are functionally inert [30], whereas those located inside exosomes remain functional and retain the potential to act upon mRNAs when their carrier exosomes are taken up by other cells [32]. The vast majority of research into potential miRNA biomarkers has been in the cancer field [33] with a growing body of work also exploring their role in cardiovascular disease [34]. Circulating miRNAs in the context of lentiviral infections have only been briefly explored [35–38] and it is clear that there is a complex interplay between miRNAs and HIV-1 pathogenesis [39]. There is a growing body of literature which supports the possibility that circulating miRNAs may provide prognostic biomarkers for predicting non-AIDS related mortality in individuals on long term ART.

The primary objective of this study was to identify miRNA biomarkers predictive of all-cause mortality in a group of ART treated HIV-1 infected individuals.

## Materials and Methods

### Study Details

#### Ethics

Samples analysed in this study were derived from participants in two international clinical trials, SMART (NCT00027352) [23] and ESPRIT (NCT00004978) [20], run by the INSIGHT collaboration in over 450 investigational centres between 1999 and 2009. All samples were derived from participants who provided written informed consent to use of both their data and of their stored samples for future laboratory research. All informed consents were reviewed and approved by participant site ethics review committees. The ethics for SMART, ESPRIT and this study were reviewed and approved by the UNSW Human Research Ethics Committee.

#### Study Population

Participants were selected from the control arms of the SMART [23] and ESPRIT [20] trials. All participants in these trials were HIV-1 positive. Cases were defined as those participants who died from any cause during follow up. Controls were defined as participants who were known to be alive at completion of follow up and matched for age, gender, location (continent) and randomisation date (± 3 months). To improve methodological rigour we attempted to balance the controls on the basis of specified criteria for our cases in a ratio of two controls for every case. This brings the comparisons closer to a randomised design by identifying patients who at the beginning of the observation have broadly similar characteristics for a range of prognostically important covariates. When there were more than two controls per case available two of these controls were randomly selected, however, when two matched controls were not available only one was selected (5 cases). After collection, sera were double spun and stored in sterile 1 mL aliquots at -80°C until thawed for use.

#### Clinical outcomes

Causes of death among cases was reviewed by an Endpoint Review Committee and categorised using the Coding of Death in HIV (CoDE system) [40].

### Measurement of hs-CRP, D-Dimer and IL-6

Hs-CRP, D-Dimer and IL-6 were measured by the Laboratory for Clinical Biochemistry Research at the University of Vermont (Burlington) for the SMART individuals and by the Clinical Serviced Program at the SAIC Frederick (Frederick, Maryland, USA) for the ESPRIT individuals [24].

### miRNA screen

Before performing assays on the study population the intended extraction and amplification methodology was validated using a total miRNA screen in the serum of 4 HIV-1 infected ART treated individuals and 4 healthy controls. RNA was extracted, reverse transcribed and pre-amplified according to methodologies described below. All known human miRNAs were then measured using Taqman Array Human MicroRNA Cards A + B v3 (Life Technologies) according to the manufacturer’s instructions.

### miRNA candidate biomarkers

21 miRNAs were selected based on their reported associations with human disease conditions associated with SNAEs in non HIV-infected populations or because they are encoded from a virus that is known to result in chronic infection in humans. Additionally 3 miRNAs were selected to act as reference genes and to ensure consistent RNA extraction. Full details of miRNAs and their associations are shown in Table 1.

**Table 1:** MiRNAs selected for analysis.

**miRNA**	**Reason for Inclusion**	**Reference**
**miR-16**	• Reference Gene	[41]
**U6snRNA**	• Life Technologies internal calibrator	
**U6snRNA**	• Life Technologies internal calibrator	
**Cel-miR-39**	• Spike in control for RNA extraction	
**miR-126**	• Induces CXCL12 dependent vascular protection	[32, 42–44]
	• Decreased in patients with coronary artery disease	
	• Positively associated with myocardial infarction in the Bruneck cohort	
	• Decreased in type 2 Diabetes in the Bruneck cohort	
**miR-223**	• Inversely associated with myocardial infarction in the Bruneck cohort	[43, 44]
	• Decreased in type 2 Diabetes in the Bruneck cohort	
**miR-221**	• HIV tat decreases miR-221 in HUVEC cells which increases ICAM-resulting in an increase of monocyte adhesion	[45–48]
	• Differentially regulated in a number of cancer types including colorectal non-small cell lung cancer and prostate cancer	
**miR-21**	• Decreased in type 2 Diabetes in the Bruneck cohort	[35, 43, 44, 49]
	• Increased in patients with CVD compared to aged matched non-CVD individuals while also correlating with hs-CRP and fibrinogen	
	• Induces IL-6 by binding to TLR8	
	• Increased in circulation of macaques that developed SIV related CNS disease compared to no disease	
**miR-197**	• Inversely associated with myocardial infarction in the Bruneck cohort	[43, 44, 50]
	• Decreased in type 2 Diabetes in the Bruneck cohort	
	• Increased levels correlate with hypertension and BMI possibly leading to dyslipidaemia in metabolic syndrome in metabolic syndrome	
**miR-145**	• Decreased in patients with coronary artery disease	[42]
**miR-155**	• Decreased in patients with coronary artery disease	[42, 51]
	• Increased in inflammatory liver damage	
**miR-146a**	• Upregulated in type 2 diabetic patients compared to pre-diabetic and diabetes susceptible individuals	[35, 51, 52]
	• Increased in inflammatory liver damage	
	• Increased in circulation of macaques that developed severe SIV related CNS disease compared to no disease	
**miR-122**	• Increased in inflammatory liver damage	[51, 53, 54]
	• Essential for HCV replication in liver cells	
	• Increased in hyperlipidaemia and associated with coronary artery disease	
**miR-200a**	• Increased in HCV/HIV co-infected compared to HIV mono-infected individuals	[55]
**miR-572**	• Decreased in monocytes of chronically HIV-1 infected individuals compared to LTNPs and Healthy controls	[56]
**miR-31**	• Down-regulated during HIV-1 infection and significantly associated with HIV-1 viral load and CD4+ T cell count in PBMCs	[57]
**miR-24**	• Decreased in type 2 Diabetes in the Bruneck cohort	[43, 44]
**miR-29a**	• Upregulated in type 2 diabetic patients compared to pre-diabetic and diabetes susceptible individuals	[49, 52]
	• Induces IL-6 by binding to TLR8	
**miR-370**	• Increased in hyperlipidaemia and associated with coronary artery disease	[53]
**miR-150**	• Negatively correlated with hs-CRP in atrial fibrillation patients	[58, 59]
	• Increased levels associate with lymphocyte activation	
**Let-7e**	• Let-7e correlates with IL-6 in SIV infected macaques and targets IL-6 in both macaques and humans	[35, 60, 61]
	• Increased in hypertensive patients compared to healthy controls	
**miR-134**	• Increased in patients with acute pulmonary embolism and a positive D-dimer ELISA	[62]
**EBV-miR-BART15**	• Secreted, in exosomes, from infected B-cells to non-infected cells to target the miR-223 NLRP3 binding site inhibiting IL-1B	[63]
**EBV-miR-BART1-5p**	• Transported from EBV infected B cells to non-infected cells via exosomes altering CXCL11 in these cells	[64]
**HCMV-miR-UL112**	• Increased in hypertensive patients compared to healthy controls However there is a rebuttal letter suggesting that higher CMV-miR-UL112 may be a consequence not the cause of hypertension	[60, 65]

Unless otherwise stated all studies refer to the miRNA in question in the circulation and in humans.

### Haemolysis Analysis

Free haemoglobin was measured in all of the serum samples using a Roche Diagnostics Modular P analyser (Hoffman-La Roche. Basel, Switzerland) according to standard methodology [66].

### RNA Extraction

In a 2mL RNase free screw top tube 1000ul of Trizol^®^ LS reagent (Life Technologies Carlsbad, CA, USA) was added to 400μl of serum. In order to increase RNA yield and to ensure extraction of GC poor miRNAs [67], 5ul of glycogen (20ng/μl) was added. This mixture was inverted end over end and then vortexed for 30 seconds before being incubated at room temperature for a further 10 minutes. 3.5ul of Cel-miR-39 (1.6x10^8^ copies/μl) (Qiagen, Venlo, Limburg, Netherlands) was then added to the mixture. Following this 200μl of chloroform was added and the mixture was shaken vigorously for 20 seconds before being incubated at RT for 15 minutes. Afterwards the mixture was centrifuged in a Heraeus Fresco microcentrifuge (Thermo Scientific, Waltham, MA USA) at 12,000g for 15 minutes at 4°C. The aqueous phase was removed into a new 2mL tube before 1000μl of 100% isopropanol was added. The resulting mixture was vortexed for 5 seconds and incubated at RT for 10 minutes before centrifugation at 12,000g for 8 minutes at 4°C. The supernatant was removed and 1000μl of cold 75% ethanol was added to the pellet and the tubes were inverted 5 times. This mixture was centrifuged at 7500g for 5 minutes at 4°C. The supernatant was carefully removed and the pellet was allowed to air dry. The dried pellet was eluted into 25μl of RNase free H_2_O and stored at -80°C.

### Reverse Transcription

RNA was reverse transcribed using the Taqman MicroRNA Reverse Transcription Kit (Life Technologies) according to manufacturer’s instructions. One reaction of the reverse transcription mastermix for both the Taqman array cards and 384 well PCRs consisted of 6μl of a custom RT primer pool, 0.3μl of dNTPs, 3ul of reverse transcriptase, 1.5μl of 10xRT buffer, 0.19 RNase inhibitor and 1.01μl of nuclease free water. In 96 well PCR plates 12μl of the RT mastermix was combined with 3μl of sample RNA. Samples were run on Veriti Thermocycler (Life Technologies) for 30 minutes at 16°C followed by 30 minutes at 42°C and 5 minutes at 85°C. The resulting cDNA was stored at -80°C.

### Preamplification

A single reaction of the pre-amplification mastermix used for both Taqman array cards and 384 well PCRs consisted of 12.5μl of 2x Pre-amplification mastermix (Life Technologies) 3.75μl of custom pre-amplification primers and 6.25ul nuclease free water. In 96 well plates 22.5μl of the pre-amplification master-mix was combined with 2.5μl of cDNA from the RT reaction according to the manufacturer’s instructions. Samples were run on a Veriti Thermocycler (Life Technologies) for 95°C for 10 minutes, 2 minutes at 55°C, 2 minutes at 72°C followed by 12 cycles of (15 seconds at 95°C and 4 minutes at 60°C) and finally 99.9°C for 10 minutes. The resulting pre-amplification product was diluted 1:8 with 0.1x TE buffer and stored at -80°C.

### RTqPCR

The 24 miRNAs chosen for analysis were measured, in duplicate according to manufacturer’s instructions, using custom designed taqman array cards. Briefly 100μl of diluted pre-amplified product was combined with 100μl of 2x Gene Expression Mastermix and added to the wells of the Array Card. The array cards were then centrifuged for 2 x 1 minute spins at 331g using a Heraeus Multifuge 3S-R (Thermo-Scientific) before being sealed using the Array Card Staker/Sealer (Life Technologies). Assays were repeated for the 5 human miRNAs that showed non-specific amplification, using the custom Taqman array cards, on 384 well PCR plates. Each individual PCR reaction on the 384 well plates consisted of 5μl of 2x Gene expression mastermix, 0.5μl of probe/primer mix (life Technologies), 4μl of nuclease free water and 0.5ul of the diluted pre-amplification product. Both the array cards and the 384 well plates were run on a Quantstudio 7 RTqPCR machine (Life Technologies). MiR-16 was run on both the array cards and 384 well plates and acted as a reference gene.

### Statistical Analysis

#### Power Calculations

Power calculations were conducted *a priori* and indicated that for all-cause mortality, using a binary miRNA split at the median value in the control group, the study had 80% power to detect an odds ratio of 2.0. The study was less well powered for the analyses of cardiovascular and malignancy related mortality and these analyses are viewed as more exploratory. The power of the analyses is best interpreted by the 95% confidence intervals given for all effect estimates.

#### miRNA Quantitation

All 21 candidate biomarker miRNAs were measured in duplicate in the serum of the cases and controls and normalised to miR-16 expression. Significant non-specific amplification was observed for miRs -29a, -197, -572, -146a and -155 using the Taqman Array Cards (data not shown). Therefore, the assays for these miRNAs were repeated using a reverse transcription and pre-amplification primer pool consisting of only the 5 failed miRNAs primers as well as the miR-16 primers. The resulting pre-amplified cDNA was analysed by RTqPCR in 384 well plates. HCMV-miR-UL112 PCR primers were suspected to be interacting with the human miRNA primers during the pre-amplification step, causing non-specific amplification and were therefore excluded (data not shown). Baseline miRNA relative abundance was measured and defined as the Ct (the point at which the reporter fluorescence becomes greater than the threshold and is considered a real signal) of the target miRNA normalised to miR-16 (Ct of miRNA of interest–CT of miR-16). These values were then summarised for cases and controls as medians and interquartile ranges (IQRs). EBV-miR-BART15 and EBV-miR-BART-1-5P were only detected reliably (<33 cycles) in 5 and 12 individuals respectively and were therefore excluded from further statistical analysis. One individual (a control) showed no amplification in any of the miRNAs tested and was therefore excluded from the analysis.

#### miRNA association with mortality (all-cause)

Associations of miRNAs with all-cause mortality were explored using conditional logistic regression analyses, matching cases with controls. Initial unadjusted analyses assessed the increased odds of mortality per unit (log10) increase in relative abundance of each miRNA. Subsequent analyses were based on splitting each miRNA into quartiles based on the values among controls. The following analyses were performed; first, unadjusted analyses assessed the odds of death in each quartile compared to the lowest quartile as reference group; Second, analyses adjusted for age, race (white, black, other), baseline CD4, ART and HIV (no ART, ART and HIV<400 copies/mL, ART and HIV>400 copies/mL), prior AIDS, hepatitis B or C co-infection, other risk factors (current smoker, diabetes, blood pressure lowering drugs, lipid lowering drugs, prior CVD, total/HDL cholesterol ratio, BMI); and third, analyses adjusted for the variables in (ii) plus CRP, d-dimer and IL-6.

#### miRNA association with mortality (cardiovascular and malignancy)

Conditional logistic regression analysis was also used to determine if miRNAs were associated with either cardiovascular or malignancy related mortality. The odds of cardiovascular or cancer death per log10 increase in each miRNA were calculated, both unadjusted and adjusted for age, CD4+ T cell count, ART and HIV RNA level, and prior AIDS. These analyses were not adjusted for all covariates because of the smaller number of events. The choice of adjustment covariates was based on those covariates that were significant in the analyses of all-cause mortality.

#### miRNA correlation with established biomarkers

Correlations between the established biomarkers (hs-CRP, D-dimer, IL-6 and CD4+ T cells) and miRNAs (normalised to miR-16) were assessed using Spearman’s non-parametric correlation coefficient with a relationship deemed significant with p<0.05. Levels of miRNAs and established biomarkers were then log10 normalised and plotted on xy scatter plots using Prism (Graphpad, La Jolla, CA, USA).

In all analyses, p-values and confidence intervals are presented unadjusted for multiple comparisons.

## Results

### Study Population

126 cases and 247 matched controls were identified from the control-arm participants of the SMART [23] and ESPRIT [20] trials. Baseline characteristics of cases and controls can be found in Table 2. Causes of death for cases are summarised in Table 3.

**Table 2:** Baseline Characteristics for Cases and Controls.

**Baseline Characteristics**	**Control (n = 247)**	**Case (n = 126)**
**Age ± SD (years)**	47.29 ± 9.62	48.29 ± 10.60
**Mean baseline CD4+ T cell count ± SD**	584.94 ± 253.11	533.865 ± 224.37
**Mean nadir CD4+ T cell count± SD**	232.79 ± 172.90	207.04 ± 159.18
**Mean BMI ± SD**	24.84 ± 4.13	24.78 ± 5.36
**Mean hs-CRP ± SD**	3.97 ± 8.56	5.62 ± 8.53
**Mean D-dimer ± SD**	0.41 ± 0.68	0.50 ± 0.54
**Mean IL-6 ± SD**	2.67 ± 2.36	5.03 ± 10.11
**%male**	82.52	82.54
**Race (%white)**	69.51	73.81
**Race (%black)**	19.92	19.84
**Race (%other)**	10.57	6.35
**Off ART (%)**	7.72	10.32
**On ART with HIV RNA ≤500 (%)**	74.39	61.90
**On ART with HIV RNA >500 (%)**	17.89	27.78
**HBV (% surface antigen positive)**	2.44	3.97
**HCV (% antibody positive)**	17.48	30.95
**Prior AIDS (% baseline positive)**	26.02	29.37
**Diabetes (% baseline positive)**	4.47	8.73
**Prior CVD (% baseline positive)**	1.63	8.73
**Lipid Lowering Drug (% baseline positive)**	17.48	18.25
**Blood Pressure Lowering Drug (% baseline positive)**	12.6	23.02
**Smoking (% smokers)**	36.75^1^	59.32^1^
**Mean total cholesterol ± SD**	198.8 ± 44.57^1^	195.2 ± 53.21^1^

^1^ Details were available for the SMART study only; control n = 117, case n = 59

**Table 3:** Causes of Death for the SMART and ESPRIT cohorts.

	SMART		ESPRIT		Overall	
Cause of death	Number	%	Number	%	Number	%
Opportunistic disease (AIDS)	3	5.1	3	4.5	6	4.8
CVD + unwitnessed/sudden death	20	33.9	24	35.8	44	35
Hepatic disease	3	5.1	10	14.9	13	10.3
Renal disease	2	3.4	0	0	2	1.6
Infection (excluding OD and hep B/C)	5	8.5	2	3	7	5.6
Non-AIDS malignancy	12	20.3	11	16.4	23	18.3
Trauma	3	5.1	6	9	9	7.1
Substance abuse/intoxication	6	10.1	4	6	10	7.9
Suicide	2	3.4	2	3	4	3.2
Other	3	5.1	5	7.4	8	6.2
Total	59	100	67	100	126	100

### Sample Quality Control

In order to validate the use of miR-16 as a reference gene, the extent of haemolysis was assessed by measuring free haemoglobin in all samples prior to analysis. Less than 8% of all samples showed any levels of haemolysis, with 75% of these haemolysed samples exhibiting very low levels of haemolysis (haemoglobin between 10 and 20mg/dL). Additionally, the distribution of samples with high levels of haemoglobin level (above > 10mg/dL) was similar between the two groups (cases: 41% of haemolysed samples; controls: 59%). Due to the relatively small numbers of haemolysed samples and their even spread across cases and controls, it was highly unlikely that these samples would affect downstream analysis, so no samples were excluded due to haemolysis.

CEL-miR-39 spike in control was added to the samples prior to RNA extraction to ensure consistent extraction. RTqPCR results showed this miRNA was expressed stably throughout all the samples (S1 Fig) thus validating the extraction process.

### miRNA association with all-cause mortality

The main aim of the study was to determine if any of the individual miRNAs measured (S1 Table) showed an association with all-cause mortality in ART treated individuals. Unadjusted logistic regression analysis failed to find any significant associations between any of the miRNAs analysed and all-cause mortality (Table 4). As not all miRNAs were detected in all samples the number of cases and controls varies for each miRNA tested. Pre-specified adjusted analyses based on miRNAs separated into quartiles also failed to find any associations between the miRNAs tested and all-cause mortality (S2 Table). Some miRNAs reached significance when comparing individual quartiles; however, this was always limited to a single quartile. Overall, none of these miRNAs was associated with all-cause mortality.

**Table 4:** Associations of all-cause mortality with miRNA levels.

**miRNA**	**n, Median (IQR)**	**Univariate OR (95% CI), p value**
**miR-126**		
Control	246, 3.09 (2.21, 3.69)	
Case	126, 2.71 (2.25, 3.68)	0.98 (0.79, 1.20), 0.82
**Let-7e**		
Control	245, 6.71 (5.91, 7.42)	
Case	125, 6.63 (5.99, 7.47)	1.05 (0.88, 1.27), 0.57
**miR-21**		
Control	246, 6.34 (5.34, 7.07)	
Case	126, 6.04 (5.11, 6.87)	0.91 (0.77, 1.08), 0.28
**miR-24**		
Control	246, 4.64 (3.88, 5.42)	
Case	125, 4.46 (3.71, 5.16)	0.99 (0.82, 1.18), 0.88
**miR-122**		
Control	246, 6.60 (4.91, 7.70)	
Case	125, 6.05 (4.39, 7.83)	0.94 (0.86, 1.04), 0.23
**miR-134**		
Control	246, 8.65 (7.24, 10.16)	
Case	126, 8.74 (7.34, 10.41)	1.07 (0.98, 1.16), 0.15
**miR-145**		
Control	246, 9.75 (8.23, 10.69)	
Case	125, 9.68 (8.79, 10.47)	1.10 (0.97,1.26), 0.14
**miR-200a**		
Control	218, 15.01 (13.54, 16.20)	
Case	116, 14.62 (13.24, 15.93)	0.93 (0.83, 1.04), 0.18
**miR-150**		
Control	246, 4.55 (3.25, 5.58)	
Case	126, 4.48 (4.48, 3.35)	1.07 (0.94, 1.23), 0.31
**miR-221**		
Control	246, 8.14 (6.90, 9.28)	
Case	125, 8.21 (7.32, 9.08)	1.10 (0.96,1.25), 0.16
**miR-223**		
Control	246, -0.96, (-1.79, -0.15)	
Case	126, -1.02 (-2.08, -0.34)	0.97 (0.81,1.15), 0.75
**miR-31**		
Control	175, 15.69 (14.19, 16.87)	
Case	96, 15.33 (13.69, 16.88)	0.91 (0.80, 1.04), 0.17
**miR-370**		
Control	238, 11.45 (9.90, 12.75)	
Case	115, 11.28 (10.20, 12.92)	1.03 (0.94, 1.13), 0.55
**miR-29a**		
Control	223, 5.43 (4.14, 6.58)	
Case	114, 5.11 (4.22, 6.10)	93 (0.81, 1.07), 0.31
**mir-146a**		
Control	237, 0.87 (-0.31, 2.13)	
Case	120, 1.06 (-0.15, 1.90)	1.01 (0.90, 1.13), 0.91
**miR-197**		
Control	236, 5.62 (4.23, 7.14)	
Case	116, 5.66 (4.40, 6.60)	1.02 (0.91, 1.13), 0.78
**miR-155**		
Control	221, 6.55 (5.48, 7.85)	
Case	111, 6.63 (5.19, 7.39)	0.95 (0.86, 1.05), 0.31
**miR-572**		
Control	202, 13.34 (10.75, 15.24)	
Case	96, 13.07 (11.74, 14.46)	0.98 (0.89, 1.07), 0.62

### miRNA association with cause-specific mortality (cardiovascular and malignancy)

Logistic regression analyses were also performed to determine if there was any association between miRNAs and either cardiovascular or malignancy related deaths. These two clinical outcomes formed the majority of the deaths in the cases (35% and 18% respectively) and were the only ones that had a large enough sample size for individual statistical analysis. However, again, no association with mortality was found (S3 and S4 Tables).

### miRNA correlation with established biomarkers

**Fig 1:**
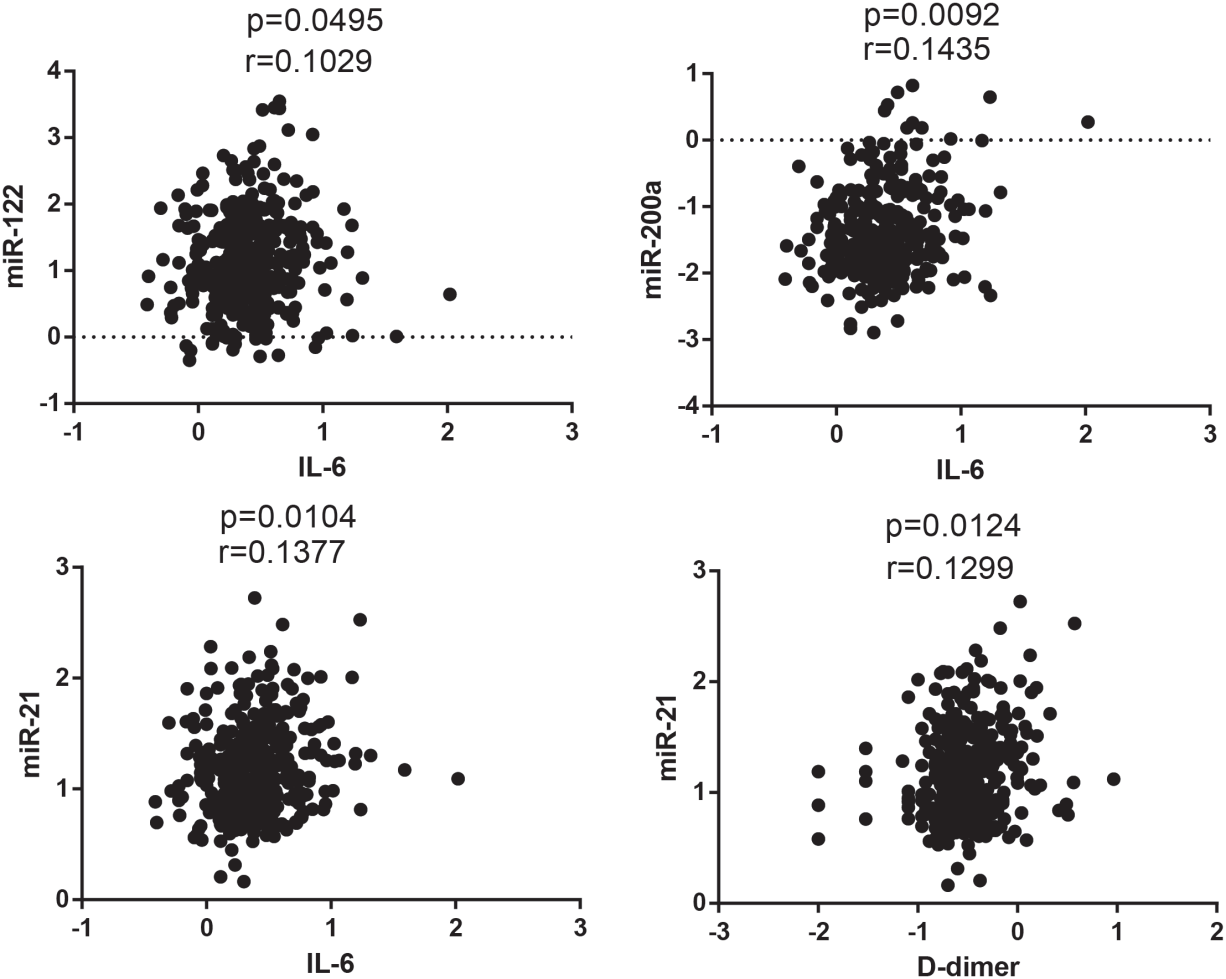
miRNA Correlations with IL-6 and D-dimer. MiR-16 normalised miRNA values were plotted against levels of established SNAE biomarkers (Il-6, D-dimer and hs-CRP). Both miRNA and biomarker levels were log transformed and correlated using the nonparametric Spearman’s Correlation co-efficient. Data was considered significant with a p value < 0.05. MiRs -200a, -122 and -21 all showed correlation with Il-6. MiR-21 additionally showed correlation with D-dimer. None of the miRNAs showed any correlation with hs-CRP.

Increased levels of the inflammatory markers IL-6 and hs-CRP and the coagulation marker D-dimer have been shown to be associated with increased morbidity and mortality in ART treated HIV-1 infected individuals [24, 26]. However, the exact mechanism by which the inflammatory and coagulation pathways are triggered and how these pathways associate with SNAEs is not clear. If not predictive of the adverse outcomes themselves the miRNAs may still provide some insight as to the mechanism by which the established biomarkers associate with SNAEs. Spearman’s correlations comparing hs-CRP, D-dimer and IL-6 and the individual miRNAs were analysed. While no miRNA showed any significant correlation with hs-CRP, three miRNAs, miR -21 (r = 0.13), -122 (r = 0.10) and -200a (r = 0.14), showed significant correlations with IL-6 and miR-21 (r = 0.13) showed an additional correlation with D-dimer (Fig 1).

**Fig 2:**
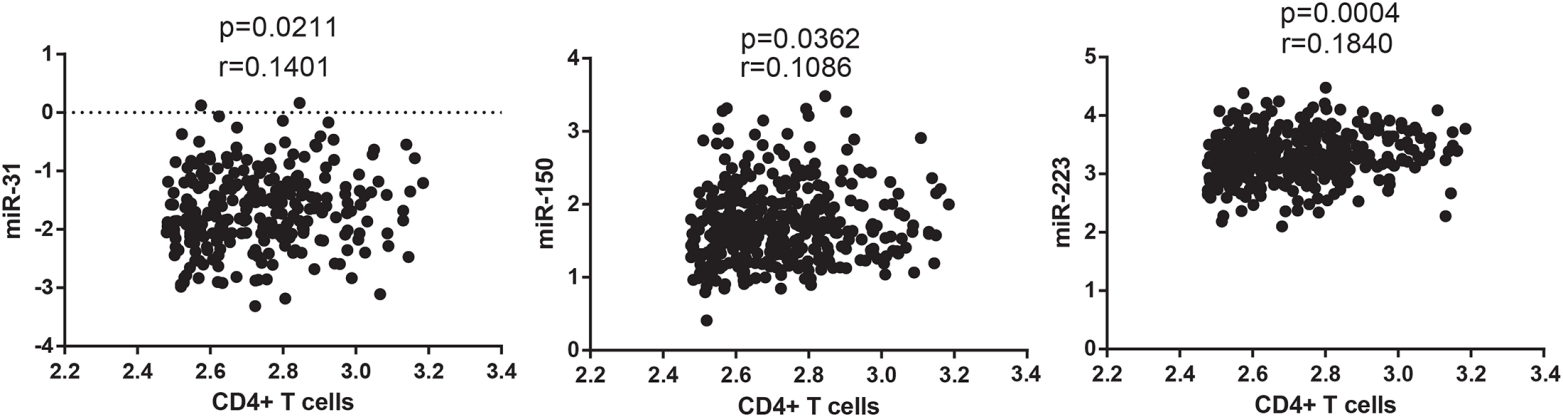
miRNA Correlations with baseline CD4+ T cell number. MiR-16 normalised miRNA values were plotted against CD4+ T cells measured at baseline. Both miRNA and CD4+ T cell count were log transformed and correlated using the nonparametric Spearman’s Correlation co-efficient. Data was considered significant with a p value < 0.05. Only MiRs -31, -150 and -223 showed significant correlation with CD4+ T cell number.

CD4+ T cell counts have also previously shown significant associations with SNAEs [10, 25]. Of the 21 miRNAs measured three, miR-31 (r = 0.14), -150 (r = 0.11)and -223 (r = 0.18), significantly correlated with the CD4+ T cell count taken at the same time as the serum samples from which the miRNAs were measured (Fig 2). Additionally, expression of miRs -31 (r = 0.12), -150 (r = 0.10), -223 (r = 0.13) as well as miR-145 (r = 0.13) correlated with nadir CD4+ T cell counts (S2 Fig).

## Discussion

Recent research in non HIV-1 infected individuals has suggested that miRNAs are either predictive of or associated with a wide variety of human disease conditions, including those that contribute to the increased morbidity and mortality seen in ART treated individuals. However, no study has explored their potential to predict mortality in ART treated HIV-1 infected individuals. As SNAEs represent a considerable burden of disease in the HIV-1 infected population we decided to explore the associations between both all-cause mortality and SNAEs, that resulted in death (specifically cardiovascular and malignancy related death), and a selected panel of circulating miRNAs. What sets this study apart from the majority of other studies into circulating miRNAs is that for every case there were carefully chosen matched controls. This is the first example of this methodology being employed to explore the predictive potential of miRNAs in an HIV-1 context. Importantly this study is one of few that utilises matched controls in all of miRNA biomarker research. The miRNAs that were tested have been shown, in other studies, to significantly associate with human disease conditions that contribute to SNAEs. However, when evaluated in this set of ART treated HIV-1 infected cases and controls they showed no associations with all-cause mortality. Additionally no associations between miRNA and death were observed when the cardiovascular and malignant deaths were analysed individually. This is despite many of these miRNAs showing clear associations with these two disease conditions in the literature (Table 1) Since no one cancer dominated the malignancy deaths it was difficult to select miRNAs that associated with general malignancy, as the majority of studies into circulating miRNAs in malignant disease focused on one particular cancer type. However, the majority of miRNAs selected have a clear association with cardiovascular disease (Table 1). Yet it is unclear whether our results indicate that these miRNAs have no association with cardiovascular disease in the general population as our study focused on ART-treated HIV-1 infected individuals. The lack of association may also be due to insufficient power of the analysis into cardiovascular related deaths.

While there were no associations with mortality a subset of the miRNAs were correlated with biomarkers that are thought to contribute to pathogenic pathways of SNAEs including levels of D-dimer (miR-21), IL-6 (miR-21, miR-122 and miR-200a) and CD4+ T cell count, both nadir (miR-145, - 31, -150 and -223) and baseline (miRs -31, -150 and -223). However, these correlations are quite weak. Moreover, it is unclear from the results in this study whether the miRNA changes observed are happening up or downstream of the activation of the biomarker with which they correlate. Overall, the correlations we observed between the miRNAs and established biomarkers are not completely discordant with results from other studies. IL-6 has been linked with circulating levels of miR-21 in the past [49] and three of the miRNAs that associate with CD4+ T cell count in our study (miRs -31, -150 and -223) have been linked previously with HIV-1 pathogenesis [37, 38, 57, 68]. Indeed correlations between CD4+ T cell count and mir-31 and mir-150 during HIV-1 infection have been observed previously, albeit in cell associated miRNAs derived from PBMCs [37, 57] rather than in serum as we observed here, providing support for the validity of the results observed here.

### Limitations

While the circulating miRNAs analysed here are clearly not useful biomarkers for mortality on ART this does not rule out all microRNAs. The miRNAs tested are only a small subset of the few hundred miRNAs that have been found to be present in the circulation. It is possible that other circulating or cell associated miRNAs are predictive of mortality in ART treated HIV-1 infected individuals. However, most approaches to analysing total miRNAs rely on the use of small numbers of individual samples, or pool material from several individuals to establish possible associations which are then confirmed in a larger sample size. This approach allows the measurement of a larger selection of miRNAs but may miss miRNAs that exhibit subtle yet significant changes due to the low sample size required to make such screens financially feasible. It was therefore decided to focus on miRNAs that already have proven associations with the disease conditions that contribute to SNAEs (Table 1) and measure these in the cases and controls chosen. Additionally as our study focused on the associations of miRNAs with mortality (all-cause, cardiovascular and malignancy) we are unable to comment on potential miRNA associations with individual disease conditions, such as Non-Hodgkin’s Lymphoma or CNS disease, in HIV-1 infected individuals [35, 38]. Also, as the individuals analysed in this study were not selected based on duration of ART, the data for which was unavailable, or HIV-1 disease stage we are unable to provide further commentary to recent studies analysing associations between certain circulating miRNAs and HIV-1 disease progression or an individual’s response to therapy [36, 37].

Another possible reason as to why this study showed no associations with mortality is that our methodology does not discriminate between miRNAs bound to AGO protein and exosome associated miRNAs. As the same miRNA could be present both bound to AGO and located in exosomes, due to its release by different cell types, it is possible that a miRNA of one particular source could be differentially expressed but its signal is masked by miRNAs of the alternate source. Similarly the use of serum rather than individual cell types allows us to measure just the equilibrium of the release and uptake or degradation of miRNA in the serum. Serum analysis does not distinguish between miRNAs of different cellular sources which may miss perturbations of production from one source if these are balanced by changes in the production or uptake of the same miRNA by another source. However serum is easily accessible and much more likely to provide a viable substrate for a biomarker than purified cell populations.

### Conclusion

Recent studies have suggested circulating miRNAs can act as effective biomarkers for a number of disease conditions including cardiovascular, renal, hepatic and malignancies. However when evaluating a set of these miRNAs in a carefully chosen set of HIV-1 infected cases and controls from two large long-term HIV trials with clear clinical outcomes (death on ART) no associations with death were observed. This is despite some of these miRNAs correlating with established SNAE biomarkers. The associations with established biomarkers suggest that some of the miRNAs may indeed show associations if measured closer to the events. However this association may not come soon enough and as an early predictor of adverse outcomes, on ART, miRNAs do not appear to be effective biomarkers.

## Supporting Information

S1 Fig Distribution of CEL-miR-39. CEL-miR-39 was spiked in during the RNA extraction process to ensure consistent extraction. CEL-miR-39 expression was both consistently and highly expressed (mean 18.5, SD 1.5) indicating a consistent extraction process. CEL-miR-39 expression is represented as a raw Ct value.
(TIF)

S2 Fig miRNA Correlations with nadir CD4+ T cell number. MiR-16 normalised miRNA values were plotted against nadir (lowest recorded) CD4+ T cell count. Both miRNA and CD4+ T cell number were log transformed and correlated using the nonparametric Spearman’s Correlation co-efficient. Data was considered significant with a p value < 0.05. Only MiRs -31, -245, -150 and -223 showed significant correlation with nadir CD4+ T cell number.
(TIF)

S1 Table Relative Expression of miRNA in both Cases and Controls. (DOCX)

S2 Table Risk of all-cause mortality associated with MiRNA levels at study entry. Cut-offs used based on quartiles in controls. Adjusted analyses also adjusted for age, race, CD4+ T cell count, ART and HIV status, prior AIDS, HBV, HCV, prior diabetes, blood pressure lowering treatment, lipid lowering treatment, prior CVD.
(DOCX)

S3 Table Risk of cardiovascular death. Odds ratios (OR) are per unit increase of each miRNA. The adjusted analyses are adjusted for age, CD4+ T cell count, ART and HIV status and prior AIDS. Not adjusted for all covariates due to small numbers of cases. Covariates to adjust for chosen from preliminary analyses in the cardiovascular deaths and matched controls.
(DOCX)

S4 Table Risk of Cancer Death. Odds ratios (OR) are per unit increase of each miRNA. The adjusted analyses are adjusted for age, CD4+ T cell count, ART and HIV status and prior AIDS. Not adjusted for all covariates due to small numbers of cases. Covariates to adjust for chosen from preliminary analyses in the cancer deaths and matched controls.
(DOCX)
