# Supplementary figures and images for: Circulating microRNAs in Sera Correlate with Soluble Biomarkers of Immune Activation but Do Not Predict Mortality in ART Treated Individuals with HIV-1 Infection: A Case Control Study
Source: PLoS One. 2015 Oct 14;10(10):e0139981. doi: 10.1371/journal.pone.0139981 (PMC4605674; doi:10.1371/journal.pone.0139981)

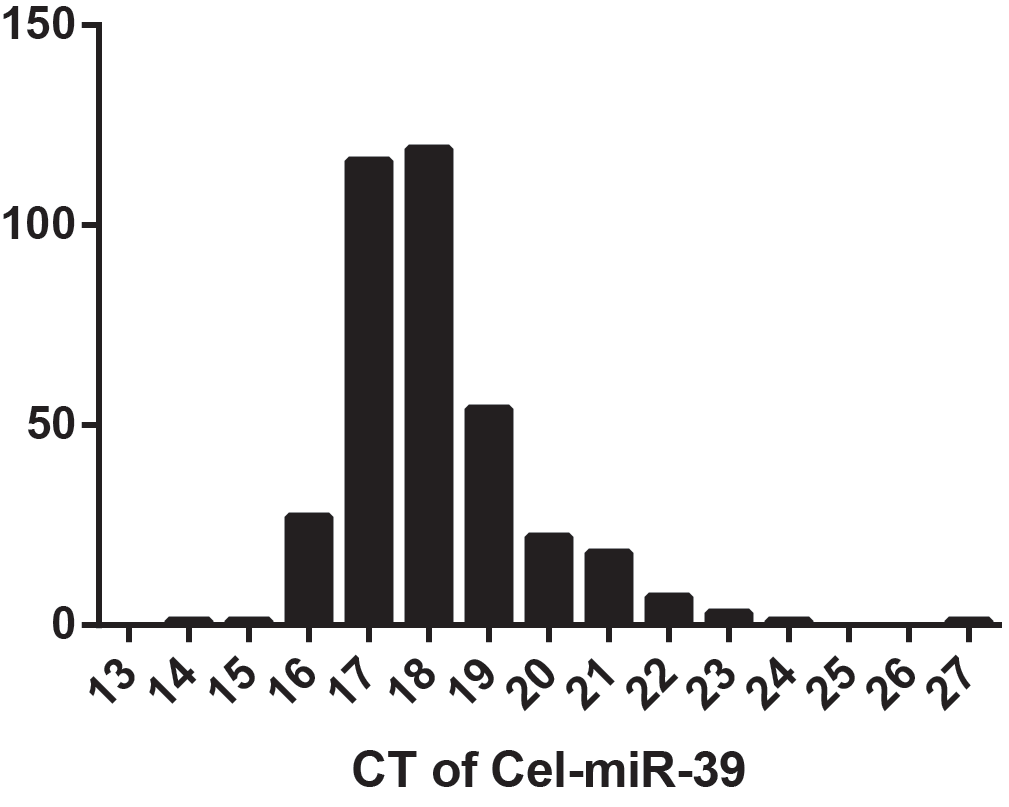

Supplement: S1 Fig — CEL-miR-39 was spiked in during the RNA extraction process to ensure consistent extraction. CEL-miR-39 expression was both consistently and highly expressed (mean 18.5, SD 1.5) indicating a consistent extraction process. CEL-miR-39 expression is represented as a raw Ct value. (TIF) [file pone.0139981.s001.tif]

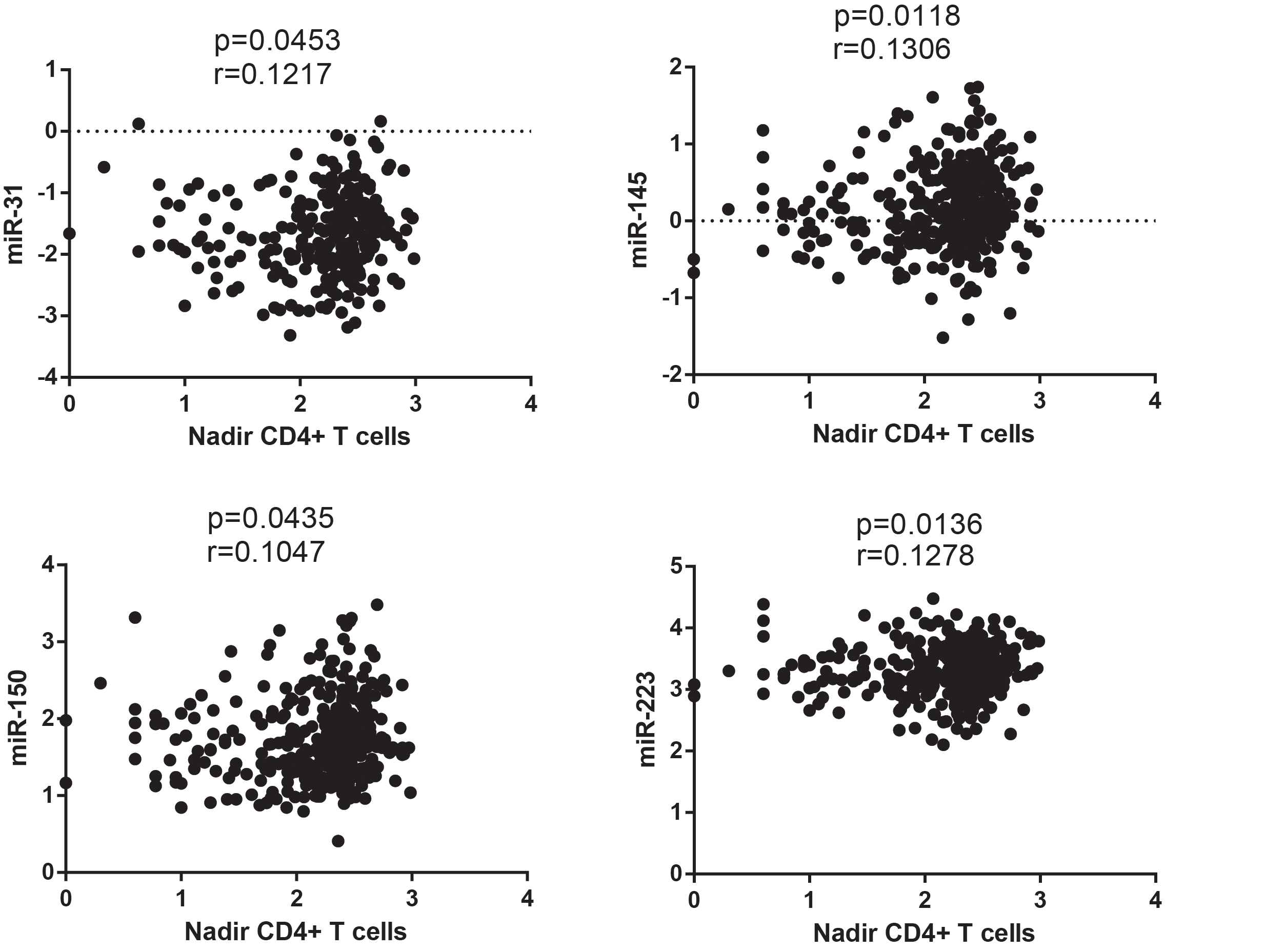

Supplement: S2 Fig — MiR-16 normalised miRNA values were plotted against nadir (lowest recorded) CD4+ T cell count. Both miRNA and CD4+ T cell number were log transformed and correlated using the nonparametric Spearman’s Correlation co-efficient. Data was considered significant with a p value < 0.05. Only MiRs -31, -245, -150 and -223 showed significant correlation with nadir CD4+ T cell number. (TIF) [file pone.0139981.s002.tif]
