# Supplementary material for: Circulating microRNAs in Sera Correlate with Soluble Biomarkers of Immune Activation but Do Not Predict Mortality in ART Treated Individuals with HIV-1 Infection: A Case Control Study
Source: PLoS One. 2015 Oct 14;10(10):e0139981. doi: 10.1371/journal.pone.0139981 (PMC4605674; doi:10.1371/journal.pone.0139981)
Supplement: S1 Table — (DOCX) [file pone.0139981.s003.docx]

Supplemental Table 1 Relative Expression of miRNA in both Cases and Controls

|  | Cases ± SD | Controls ± SD |
| --- | --- | --- |
| miR-126 | 2.94 ± 1.09 | 2.98 ± 1.03 |
| Let-7e | 6.75 ± 1.23 | 6.68 ± 1.14 |
| miR-21 | 5.96 ± 1.29 | 6.11 ± 1.36 |
| miR-24 | 4.54 ± 1.23 | 4.57 ± 1.19 |
| miR-122 | 5.98 ± 2.57 | 6.28 ± 2.26 |
| miR-134 | 8.85 ± 2.22 | 8.48 ± 2.60 |
| miR-145 | 9.70 ± 1.52 | 9.44 ± 1.78 |
| miR-200a | 14.37 ± 2.23 | 14.70 ± 2.03 |
| miR-150 | 4.40 ± 1.61 | 4.21 ± 1.78 |
| miR-221 | 8.26 ± 1.48 | 8.02 ± 1.75 |
| miR-223 | 1.04 ± 1.40 | -0.99 ± 1.26 |
| miR-31 | 15.37 ± 2.37 | 15.59 ± 2.03 |
| miR-370 | 11.45 ± 2.31 | 11.34 ± 2.57 |
| miR-29a | 5.08 ± 1.62 | 5.24 ± 1.84 |
| miR-146a | 0.82 ± 1.87 | 0.76 ± 2.12 |
| miR-197 | 5.47 ± 1.92 | 5.45 ± 2.29 |
| miR-155 | 6.11 ± 2.44 | 6.26 ± 2.39 |
| miR-572 | 12.65 ± 2.83 | - 1. 3.13 |

miRNA expression is represented as DeltaCt (miRNA of interest- miR-16)
