# Supplementary material for: Circulating microRNAs in Sera Correlate with Soluble Biomarkers of Immune Activation but Do Not Predict Mortality in ART Treated Individuals with HIV-1 Infection: A Case Control Study
Source: PLoS One. 2015 Oct 14;10(10):e0139981. doi: 10.1371/journal.pone.0139981 (PMC4605674; doi:10.1371/journal.pone.0139981)
Supplement: S2 Table — Cut-offs used based on quartiles in controls. Adjusted analyses also adjusted for age, race, CD4+ T cell count, ART and HIV status, prior AIDS, HBV, HCV, prior diabetes, blood pressure lowering treatment, lipid lowering treatment, prior CVD. (DOCX) [file pone.0139981.s004.docx]

Supplemental Table 2 Risk of all-cause mortality associated with MiRNA levels at study entry

| MiRNA | Analysis | <25^th^ percentile | 25^th^-49^th^ percentile | OR | p | 50^th^-74^th^  percentile | OR2 | *p* | ≥75^th^ percentile | OR4 | p | OR associated with one IQR higher miRNA level | OR (95% CI) | | p |
| --- | --- | --- | --- | --- | --- | --- | --- | --- | --- | --- | --- | --- | --- | --- | --- |
| M126 | Number | 29/62 |  | 49/61 |  |  | 19/62 |  |  | 29/61 |  |  |  |  | |
|  | Univariate | 1 |  | 1.67 | 0.087 |  | 0.66 | *0 0.241* |  | 1.02 | 0.948 |  | 0.92 (0.76, 1.12) | 0.39 | |
|  | Adjusted | 1 |  | 1.4 | 0.334 |  | 0.59 | *0.203* |  | 0.89 | 745 |  | 0.89 (0.71, 1.11) | 0.289 | |
|  |  |  |  |  |  |  |  |  |  |  |  |  |  |  | |
| M7e | Number | 32/64 |  | 37/59 |  |  | 24/63 |  |  | 32/59 |  |  |  |  | |
|  | Univariate | 1 |  | 1.28 | 0.42 |  | 0.78 | *0.436* |  | 1.1 | 0.744 |  | 0.98 (0.81, 1.18) | 0.823 | |
|  | Adjusted | 1 |  | 0.98 | 0.952 |  | 0.48 | *0.067* |  | 0.94 | 0.854 |  | 0.92 (0.74, 1.14) | 0.444 | |
|  |  |  |  |  |  |  |  |  |  |  |  |  |  |  | |
| M21 | Number | 37/61 |  | 37/62 |  |  | 28/62 |  |  | 24/61 |  |  |  |  | |
|  | Univariate | 1 |  | 0.93 | 0.821 |  | 0.72 | *0.299* |  | 0.62 | 0.158 |  | 0.84 (0.69, 1.04) | 0.105 | |
|  | Adjusted | 1 |  | 0.9 | 0.767 |  | 0.58 | *0.149* |  | 0.66 | 0.293 |  | 0.84 (0.66, 1.06) | 0.143 | |
|  |  |  |  |  |  |  |  |  |  |  |  |  |  |  | |
| M24 | Number | 40/63 |  | 30/61 |  |  | 33/61 |  |  | 22/61 |  |  |  |  | |
|  | Univariate | 1 |  | 0.8 | 0.481 |  | 0.84 | *0.584* |  | 0.57 | 0.089 |  | 0.85 (0.70, 1.04) | 0.122 | |
|  | Adjusted | 1 |  | 0.75 | 0.44 |  | 0.81 | *0.573* |  | 0.4 | 0.024 |  | 0.78 (0.62, 0.99) | 0.043 | |
|  |  |  |  |  |  |  |  |  |  |  |  |  |  |  | |
| M122 | Number | 40/61 |  | 38/62 |  |  | 14/61 |  |  | 33/62 |  |  |  |  | |
|  | Univariate | 1 |  | 0.88 | 0.663 |  | 0.34 | *0.004* |  | 0.76 | 0.37 |  | 0.86 (0.70, 1.05) | 0.134 | |
|  | Adjusted | 1 |  | 0.79 | 0.51 |  | 0.27 | *0.006* |  | 0.77 | 0.489 |  | 0.88 (0.70, 1.12) | 0.297 | |
|  |  |  |  |  |  |  |  |  |  |  |  |  |  |  | |
| M134 | Number | 29/62 |  | 31/61 |  |  | 29/62 |  |  | 37/61 |  |  |  |  | |
|  | Univariate | 1 |  | 1.06 | 0.863 |  | 0.97 | *0.933* |  | 1.29 | 0.388 |  | 1.08 (0.90, 1.30) | 0.424 | |
|  | Adjusted | 1 |  | 1.37 | 0.386 |  | 0.82 | *0.609* |  | 1.36 | 0.372 |  | 1.05 (0.85, 1.29) | 0.656 | |
|  |  |  |  |  |  |  |  |  |  |  |  |  |  |  | |
| M145 | Number | 21/62 |  | 43/61 |  |  | 34/62 |  |  | 27/61 |  |  |  |  | |
|  | Univariate | 1 |  | 2.38 | 0.012 |  | 1.72 | *0.112* |  | 1.34 | 0.387 |  | 1.03 (0.85, 1.26) | 0.739 | |
|  | Adjusted | 1 |  | 2.55 | 0.02 |  | 1.59 | *0.237* |  | 1.2 | 0.66 |  | 0.99 (0.78, 1.26) | 0.943 | |
|  |  |  |  |  |  |  |  |  |  |  |  |  |  |  | |
| M200a | Number | 34/55 |  | 36/54 |  |  | 22/54 |  |  | 24/55 |  |  |  |  | |
|  | Univariate | 1 |  | 0.87 | 0.655 |  | 0.61 | *0.159* |  | 0.68 | 0.279 |  | 0.86 (0.69, 1.07) | 0.17 | |
|  | Adjusted | 1 |  | 0.82 | 0.601 |  | 0.71 | *0.432* |  | 0.7 | 0.412 |  | 0.88 (0.68, 1.16) | 0.371 | |
|  |  |  |  |  |  |  |  |  |  |  |  |  |  |  | |
| M150 | Number | 27/62 |  | 37/61 |  |  | 36/61 |  |  | 26/62 |  |  |  |  | |
|  | Univariate | 1 |  | 1.45 | 0.242 |  | 1.39 | *0.313* |  | 0.94 | 0.848 |  | 0.98 (0.79, 1.21) | 0.844 | |
|  | Adjusted | 1 |  | 1.52 | 0.256 |  | 1.22 | *0.594* |  | 0.92 | 0.841 |  | 0.95 (0.74, 1.22) | 0.705 | |
|  |  |  |  |  |  |  |  |  |  |  |  |  |  |  | |
| M221 | Number | 22/62 |  | 37/62 |  |  | 39/60 |  |  | 27/62 |  |  |  |  | |
|  | Univariate | 1 |  | 2.38 | 0.012 |  | 1.72 | *0.112* |  | 1.34 | 0.387 |  | 1.03 (0.85, 1.26) | 0.739 | |
|  | Adjusted | 1 |  | 2.05 | 0.075 |  | 2.29 | *0.039* |  | 1.07 | 0.868 |  | 1.02 (0.81, 1.29) | 0.866 | |

Cut-offs used based on quartiles in controls. Adjusted analyses also adjusted for age, race, CD4+ T cell count, ART and HIV status, prior AIDS, HBV, HCV, prior diabetes, blood pressure lowering treatment, lipid lowering treatment, prior CVD.
