# Supplementary material for: Circulating microRNAs in Sera Correlate with Soluble Biomarkers of Immune Activation but Do Not Predict Mortality in ART Treated Individuals with HIV-1 Infection: A Case Control Study
Source: PLoS One. 2015 Oct 14;10(10):e0139981. doi: 10.1371/journal.pone.0139981 (PMC4605674; doi:10.1371/journal.pone.0139981)
Supplement: S3 Table — Odds ratios (OR) are per unit increase of each miRNA. The adjusted analyses are adjusted for age, CD4+ T cell count, ART and HIV status and prior AIDS. Not adjusted for all covariates due to small numbers of cases. Covariates to adjust for chosen from preliminary analyses in the cardiovascular deaths and matched controls. (DOCX) [file pone.0139981.s005.docx]

Supplemental table 3 Risk of cardiovascular death

| MiRNA |  |  | N Median (IQR) |  | Univariate  OR, P | Adjusted  OR (95% CI) |
| --- | --- | --- | --- | --- | --- | --- |
| miR-126 | Control |  | 86 3.18 (2.60, 3.77) |  |  |  |
|  | Case |  | 44 2.80 (2.48, 3.69) |  | 0.90, 0.584 | 0.86 (0.54, 1.38) 0.530 |
|  |  |  |  |  |  |  |
| Let-7e | Control |  | 86 6.78 (6.10, 7.42) |  |  |  |
|  | Case |  | 44 6.95 (6.13, 7.49) |  | 1.11, 0.569 | 1.01 (0.65, 1.58) 0.960 |
|  |  |  |  |  |  |  |
| miR-21 | Control |  | 86 6.43 (5.29, 7.22) |  |  |  |
|  | Case |  | 44 6.11 (5.47, 6.99) |  | 1.00, 0.978 | 0.98 (0.66, 1.45) 0.920 |
|  |  |  |  |  |  |  |
| miR-24 | Control |  | 86 4.64 (3.81, 5.45) |  |  |  |
|  | Case |  | 44 4.39 (3.82, 5.20) |  | 1.01, 0.930 | 0.97 (0.64, 1.48) 0.892 |
|  |  |  |  |  |  |  |
| miR-122 | Control |  | 86 6.90 (4.55, 8.02) |  |  |  |
|  | Case |  | 44 5.95 (4.37, 8.06) |  | 0.96, 0.629 | 0.93 (0.76, 1.13) 0.462 |
|  |  |  |  |  |  |  |
| miR-134 | Control |  | 86 8.43 (6.91, 10.17) |  |  |  |
|  | Case |  | 44 8.99 (7.63, 10.72) |  | 1.10, 0.217 | 1.09 (0.90, 1.32) 0.368 |
|  |  |  |  |  |  |  |
| miR-145 | Control |  | 86 9.91 (8.13, 10.86) |  |  |  |
|  | Case |  | 44 9.91 (8.51, 10.71) |  | 1.12, 0.311 | 1.06 (0.79, 1.42) 0.695 |
|  |  |  |  |  |  |  |
| miR-200a | Control |  | 79 15.08 (13.50, 16.36) |  |  |  |
|  | Case |  | 41 15.42 (13.43, 16.72) |  | 1.02, 0.797 | 1.04 (0.80, 1.33) 0.788 |
|  |  |  |  |  |  |  |
| miR-150 | Control |  | 86 4.64 (3.30, 5.70) |  |  |  |
|  | Case |  | 44 4.47 (3.69, 5.51) |  | 1.14, 0.279 | 1.10 (0.82, 1.49) 0.531 |
|  |  |  |  |  |  |  |
| miR-221 | Control |  | 86 8.16 (6.83, 9.43) |  |  |  |
|  | Case |  | 44 8.13 (7.28, 9.16) |  | 1.09, 0.434 | 1.08 (0.81, 1.44) 0.603 |
|  |  |  |  |  |  |  |
| miR-223 | Control |  | 86 -0.97 (-2.06, -0.31) |  |  |  |
|  | Case |  | 44 -1.23 (-1.95, -0.45) |  | 1.02, 0.900 | 0.87 (0.58, 1.31) 0.502 |
|  |  |  |  |  |  |  |
| miR-31 | Control |  | 62 15.71 (13.95, 16.93) |  |  |  |
|  | Case |  | 31 15.40 (13.97, 17.26) |  | 0.92, 0.471 | 0.93 (0.69, 1.27) 0.656 |
|  |  |  |  |  |  |  |
| miR-370 | Control |  | 84 11.29 (9.75, 12.85) |  |  |  |
|  | Case |  | 42 11.49 (10.48, 12.99) |  | 1.06, 0.466 | 1.11 (0.90, 1.36) 0.343 |
|  |  |  |  |  |  |  |
| miR-29a | Control |  | 81 5.18 (4.06, 6.61) |  |  |  |
|  | Case |  | 40 5.08 (4.27, 6.08) |  | 0.98, 0.879 | 0.98 (0.71, 1.34) 0.893 |
|  |  |  |  |  |  |  |
| miR-146a | Control |  | 82 0.88 (-0.77, 2.00) |  |  |  |
|  | Case |  | 43 1.03 (-0.14, 2.08) |  | 1.11, 0.325 | 1.17 (0.90, 1.53) 0.236 |
|  |  |  |  |  |  |  |
| miR-197 | Control |  | 81 5.33 (3.63, 6.79) |  |  |  |
|  | Case |  | 42 5.81 (4.61, 6.85) |  | 1.13, 0.216 | 1.18 (0.92, 1.50) 0.190 |
|  |  |  |  |  |  |  |
| miR-155 | Control |  | 78 6.61 (5.40, 7.65) |  |  |  |
|  | Case |  | 37 6.83 (6.03, 7.78) |  | 1.01, 0.952 | 1.11 (0.81, 1.51) 0.509 |
|  |  |  |  |  |  |  |
| miR-572 | Control |  | 66 12.55 (9.87, 14.52) |  |  |  |
|  | Case |  | 35 13.47 (11.93, 14.94) |  | 1.17, 0.122 | 1.21 (0.97, 1.52) 0.086 |

Odds ratios (OR) are per unit increase of each miRNA. The adjusted analyses are adjusted for age, CD4+ T cell count, ART and HIV status and prior AIDS. Not adjusted for all covariates due to small numbers of cases. Covariates to adjust for chosen from preliminary analyses in the cardiovascular deaths and matched controls
